# Computer-aided design of PVR mutants with enhanced binding affinity to TIGIT

**DOI:** 10.1186/s12964-020-00701-y

**Published:** 2021-02-08

**Authors:** Xiaowen Zhou, Jiangfeng Du, Xiuman Zhou, Xiaoshuang Niu, Wanqiong Li, Chunxia Chen, Sifan Lv, Aijun Wu, Shanshan Gou, Yixuan Sun, Wenjie Zhai, Lu Qiu, Yuanming Qi, Wenshan Zhao, Yanfeng Gao

**Affiliations:** 1 School of Life Sciences, Zhengzhou University, Zhengzhou, 450001 China; 2 School of Pharmaceutical Sciences (Shenzhen), Sun Yat-Sen University, Shenzhen, 518107 China

**Keywords:** Cancer immunotherapy, TIGIT/PVR, Molecular dynamics, Mutagenesis, Drug design

## Abstract

**Background:**

TIGIT, as a novel immune checkpoint molecule involved in T cell and NK cell anergy, could induce the immune tolerance and escape through binding with its ligand PVR. Blockade of TIGIT/PVR is considered as a promising strategy in cancer immunotherapy. However, to facilitate the design of inhibitors targeting TIGIT/PVR, the structural characteristics and binding mechanism still need to be further studied.

**Methods:**

In this study, molecular dynamics (MD) simulations and in silico mutagenesis were used to analyze the interaction between TIGIT and its ligand PVR. Then, PVR mutants were designed and their activities were determined by using TIGIT overexpressed Jurkat cells.

**Results:**

The results suggested that the loops of PVR (CC′ loop, C′C″ loop, and FG loop) underwent a large intra-molecular rearrangement, and more hydrogen bond crosslinking between PVR and TIGIT were formed during MD simulations. The potential residues for PVR to interact with TIGIT were identified and utilized to predict high affinity PVR mutants. Through the biological activity evaluation, four PVR mutants (_PVR_S72W, _PVR_S72R, _PVR_G131V and _PVR_S132Q) with enhanced affinity to TIGIT were discovered, which could elicit more potent inhibitory effects compared with the wild type PVR.

**Conclusions:**

The MD simulations analysis provided new insights into the TIGIT/PVR interaction model, and the identified PVR mutants (_PVR_S72W, _PVR_S72R, _PVR_G131V and _PVR_S132Q) could serve as new candidates for immunotherapy to block TIGIT/PVR.

**Video Abstract**

## Background

Cytotoxic lymphocytes have been involved in the resistance of tumorigenesis and carcinogenesis process through cell-mediated immunity during cancer immunotherapy [1–3]. Cytotoxic lymphocytes, such as natural killer (NK) cells and cytotoxic T lymphocytes (CTLs), express regulatory receptors including co-stimulatory and co-inhibitory molecules, and these molecules coordinate to precisely regulate the function of cytotoxic lymphocytes [4–7]. Co-inhibitory molecules have been involved in mediating immune tolerance and escape, leading to poor therapeutic efficacy in tumor treatment [8–12].

TIGIT, with a full name of T cell immunoglobulin and ITIM domain (also known as WUCAM, Vstm3 or VSIG9), is an immunosuppressive receptor. TIGIT belongs to poliovirus receptor (PVR)/nectin family and is widely expressed on NK cells, CD8^+^ T cells, CD4^+^ T cells and Tregs cells [13–17]. Until now, the identified ligands of TIGIT contain CD155 (also known as PVR or nectin-like 5), CD112 and CD113 [15, 18, 19]. Among these ligands, PVR exhibited a higher affinity with TIGIT compared with CD112 and CD113 [19, 20]. PVR is mainly expressed on dendritic cells (DCs), T cells, B cells, macrophages and all kinds of tumor cells [14, 21–24]. Engagement of TIGIT with PVR has been involved in modulating the cytokine production of DCs and facilitating the polarization of pro-inflammatory M1 macrophages into anti-inflammatory M2 macrophages, which in turn lead to the inhibition of effector T cells and NK cells activation [13, 25–27]. The roles of PVR on normal cells are to prevent excessive immune cells activation and sustain immune homeostasis [25, 28]. However, tumor cells evade immune surveillance and induce T cells and NK cells exhaustion by overexpressing PVR [29–31]. Blocking TIGIT-PVR interaction can restore immune exhaustion of T cells and NK cells [27, 32]. Therefore, the development of TIGIT/PVR inhibitors may have potentials in cancer immunotherapy. Antibodies targeting TIGIT/PVR pathway have achieved good clinical results in cancer treatment, as so far six TIGIT-targeting antibodies were under pre-clinical or clinical trials [33, 34].

However, some intrinsic adverse effects of antibody drugs, such as off-target effects, poor tissue penetration and Fc-effector functions, could deplete lymphocytes, which limit the application of antibodies in cancer treatment [35, 36]. Therefore, the development of other types of drugs targeting TIGIT/PVR pathway is essential for cancer intervention and treatment. Studies have shown that engineered protein drugs targeting immune checkpoint molecules can avoid the disadvantages of antibodies and exhibit better antitumor efficacy [37, 38]. We reported that blockade of TIGIT/PVR by peptide could elicit strong tumor tissue penetration ability and anti-tumor immune response, even in anti-programmed cell death protein 1 (PD-1) resistant tumor model [39]. As the important role of TIGIT/PVR pathway in regulating the function of the immune cells, it's essential to develop alternative protein drugs targeting the TIGIT/PVR pathway to use individually and in combination with other treatment methods to improve the therapeutic efficacy.

The structure of TIGIT in complex with its ligand PVR has been resolved. It has been shown that both TIGIT and PVR possess three domains: extracellular domain, transmembrane region and cytoplasmic domain, and the extracellular domain has a series of glycosylation sites (Additional file 1: Fig. S1a–d). The extracellular domain of PVR is composed of D1, D2, and D3 domain, and D1 domain of PVR plays an important role in interacting with TIGIT to deliver the inhibitory signals [40]. A series of crystal structures of the extracellular domain of TIGIT and PVR was reported by using X-ray diffraction and electron microscopy (Table 1). These findings facilitate computer-aided drug design using molecular dynamics simulations for molecules which can regulate the TIGIT pathway.

**Table 1:** Specific information for the crystal structure of hPVR extracellular domain

NO	PDBID	Organism(s)	Method	Resolution (Å)	R-value	PVR length	Notations	Journal
1	3URO	Homo Sapiens	X-ray Diffraction	3.5	0.305	V30-Y242(213)	N105D, N120S, N188Q, N218Q, N237S	PNAS, 2008
2	4FQP	Homo Sapiens	X-ray Diffraction	3.6	0.248	D28-P333(306)	0	Nat Struct Mol Biol,2012
**3**	**3UDW**	**Homo Sapiens**	**X-ray Diffraction**	**2.9**	**0.251**	**D28-A143 (116)**	**0**	**PNAS,2012**
4	1DGI	Homo sapiens, Human poliovirus 1	Electron Microscopy	22	–	D28-V329(302)	0	PNAS,2000
5	1NN8	Homo sapiens, Human poliovirus 1 Mahoney	Electron Microscopy	15	–	D28-V329(302)	0	J. Virol,2003
6	3EPF	Homo sapiens, Poliovirus type 2 strain Lansing	Electron Microscopy	9	–	V30-Y242(213)	N105D, N120S, N188Q, N218Q, N237S	PNAS,2008
7	3EPD	Homo Sapiens, Human poliovirus 3	Electron Microscopy	9	–	V30-Y242(213)	N105D, N120S, N188Q, N218Q, N237S	PNAS,2008
8	3EPC	Homo sapiens, Human poliovirus 1 Mahoney	Electron Microscopy	8	–	V30-Y242(213)	N105D, N120S, N188Q, N218Q, N237S	PNAS,2008
9	3J9F	Homo sapiens, Human poliovirus 1 Mahoney	Electron Microscopy	9	–	D28-A143 (116)	0	J. Virol,2015
10	3J8F	Homo sapiens, Human poliovirus 1 Mahoney	Electron Microscopy	3.7	–	D28-P333(306)	0	J. Virol,2015

In this study, we used molecular dynamics (MD) simulations to study the interaction between TIGIT and PVR based on the resolved crystal structure (PDBID: 3UDW). We analyzed the structural moment, atomic dynamics of PVR and variations of residual pairs, which helps to identify several novel residues of PVR that potentially bind to TIGIT. Through In silico mutagenesis and cell assay with flow cytometry, we successfully predicted high affinity PVR mutants, and measured the binding affinity of the mutants and the effects of mutants on TIGIT overexpressed Jurkat cells. Finally, we identified several high affinity PVR mutants (_PVR_S72W, _PVR_S72R, _PVR_G131V, and _PVR_S132Q) with more potent inhibitory effects on TIGIT overexpressed Jurkat cells. The MD simulations of TIGIT/PVR complex may help us to understand the binding mechanism of PVR with TIGIT, and the high affinity mutants may serve as start points for the development of TIGIT-PVR inhibitors.

## Methods

### Cell culture

Human embryonic kidney 293 cells (HEK-293) were cultured in Dulbecco’s Modified Eagle’s Medium (DMEM) (GIBCO, Grand Island, USA) containing 10% Fetal Bovine Serum (FBS) (Biological Industries, USA), 100 U/mL penicillin/streptomycin (Solarbio, China). Jurkat cell line and CHO-K1 cell line were cultured in RPMI 1640 medium (GIBCO, Grand Island, USA) supplemented with 10% FBS, penicillin and streptomycin. All the cell lines were maintained in a humidified condition of 5% CO_2_ and 95% air.

### Molecular dynamics simulations

The 3D structures of hPVR alone and the PVR/TIGIT complex were retrieved from crystal structures with a PDBID of 3UDW, respectively. The 3D structures were processed by GROMACS (Version 4.6) by using OPLS/AA force field. The processed proteins were then separately solvated in two simulation systems, which were made by water cubic box with SPC water model and periodic boundary conditions (PBC = 1.0 nm) were selected. The solvated systems were neutralized with sodium ions at the physiological conditions. The molecular dynamics simulations in our study were performed in three steps. The structures were firstly relaxed through energy minimization (EM). The potential energy (E_pot_) was negative on the order of 10^–5^–10^–6^ and the maximum force (F_max_) was less than 1000 kJ/mol/nm. Next, the solvent and ions around the protein were equilibrated via two simulation phases. At the first phase, the systems were restrained under an NVT ensemble (constant Number of particles, Volume, and Temperature) and the Verlet scheme was selected for 1 ns simulation with the temperature of the system reaching a plateau at 310 K. At the second phase, equilibration of pressure was conducted under an NPT ensemble (constant Number of particles, Pressure, and Temperature) for 1 ns simulation with the pressure set to 1 bar. The Parrinello-Rahman baroslat was used to control the pressure and all bonds involving hydrogen atoms were constrained with the LINCS algorithm during the equilibration procedure. Finally, the position restraints were then released and a production run of 50 ns were performed, where the time step were set to 2 fs.

### Virtual alanine and residue scanning mutagenesis

The alanine scanning mutagenesis sequentially mutated each residue of PVR at a time to alanine and the binding energy for each mutant was calculated. Residue Scanning, also known as site-directed mutagenesis, was used to successively mutate important residues of PVR to 20 natural amino acids. In this study, Molecular operating environment (MOE) software (version 2018) was used to performed all the calculation and one site mutation was taken out for the mutagenesis. The calculations were performed by using Amber10 force field and low-mode simulation was used to generate mutants’ conformation with a maximum of 30. MM/GBVI scoring function was used to calculate the binding energy between TIGIT and PVR mutants.

### Expression of TIGIT, PVR and the mutants

The full-length human PVR and human TIGIT was cloned into the pLVX-Puro vector for constructing PVR mutants and functional experiments. All mutants were acquired under site-directed mutagenesis by using a QuickChange mutagenesis kit (Thermo Fisher, USA) and ensured via DNA sequencing. Transfections of plasmids into CHO-K1 or Jurkat cell line were carried out with PowerTrans293™ (Sixiang Biological Inc., China) according to the manufacturer’s instructions. The cells stably expressing PVR, PVR mutants or TIGIT were ascertained by fluorescent-activated cell sorting (FACS) Caliber flow cytometry, and the cells stably expressing PVR and PVR mutants were next identified using PVR antibody (A5753, ABclonal) by western blotting.

### Cell staining analysis

For cell staining, the Fc-fused protein of human TIGIT (Sino Biological Inc., China) were serially diluted from 120 to 1.875 nM. CHO-K1 cells expressing WT PVR or PVR mutants were suspended in phosphate buffered saline (PBS) and incubated with hTIGIT-Fc protein for 30 min at 4 ℃. After incubating with hTIGIT-Fc protein, cells were stained with APC conjugated anti-human IgG Fc (HP6017, Biolegend, USA) for 30 min at 4 ℃. Then, CHO-K1-PVR and CHO-K1-mutants was washed in FACS buffer (PBS with 2% FBS) and analyzed by FACS Caliber flow cytometry (BD Bioscience, USA). The cells incubated only with PE conjugated human Fc antibody were regarded as the negative controls. The mean fluorescence intensity (MFI) was used to analyze binding affinity of WT and mutant PVR to its ligand TIGIT.

### Co-culture assay

CHO-K1, CHO-K1-hPVR and CHO-K1-mutant cells were seeded into 24 well plates at 1 × 10^5^ cells/well, respectively. Jurkat-hTIGIT cells (2 × 10^5^ cells/well) were co-cultured with CHOK1, CHOK1-hPVR or CHO-K1-mutant cells in the presence of 1 μg/mL anti-human CD3 and 0.5 μg/mL anti-human CD28 for 48 h at 37 ℃. The protein transport inhibitor (eBioscience) were added at the last 4 h before the end of co-culture. For cell staining, Jurkat-hTIGIT cells collected after co-culture were incubated with anti-human TIGIT PE (MBSA43, eBioscience) for 30 min at 4 ℃ and then fixated and permeabilized. Afterwards, permeabilized cells were stained with anti-human IL-2 APC (MQ1-17H12, Belegend) or isotype control for 30 min at 4 ℃. Jurkat cells was washed in FACS buffer (PBS with 2% FBS) for flow cytometry analysis.

### Statistical analysis

For binding and coculture assay, statistical differences were analyzed by GraphPad Prism 7.04. Statistical significance among different groups was calculated by Student’s *t*-test. For correlation analysis, the data were calculated by R language. The Pearson product-moment correlation coefficient represented correlation between protein expression and binding affinity. The statistically significant values were considered as follows: *P < 0.05, **P < 0.01, and ***P < 0.001. Values of *P* < 0.05,* P* < 0.01, and *P* < 0.001 were considered as statistically significant.

## Results

### Structural dynamics and hydrogen bond crosslinking of PVR

**Fig. 1:**
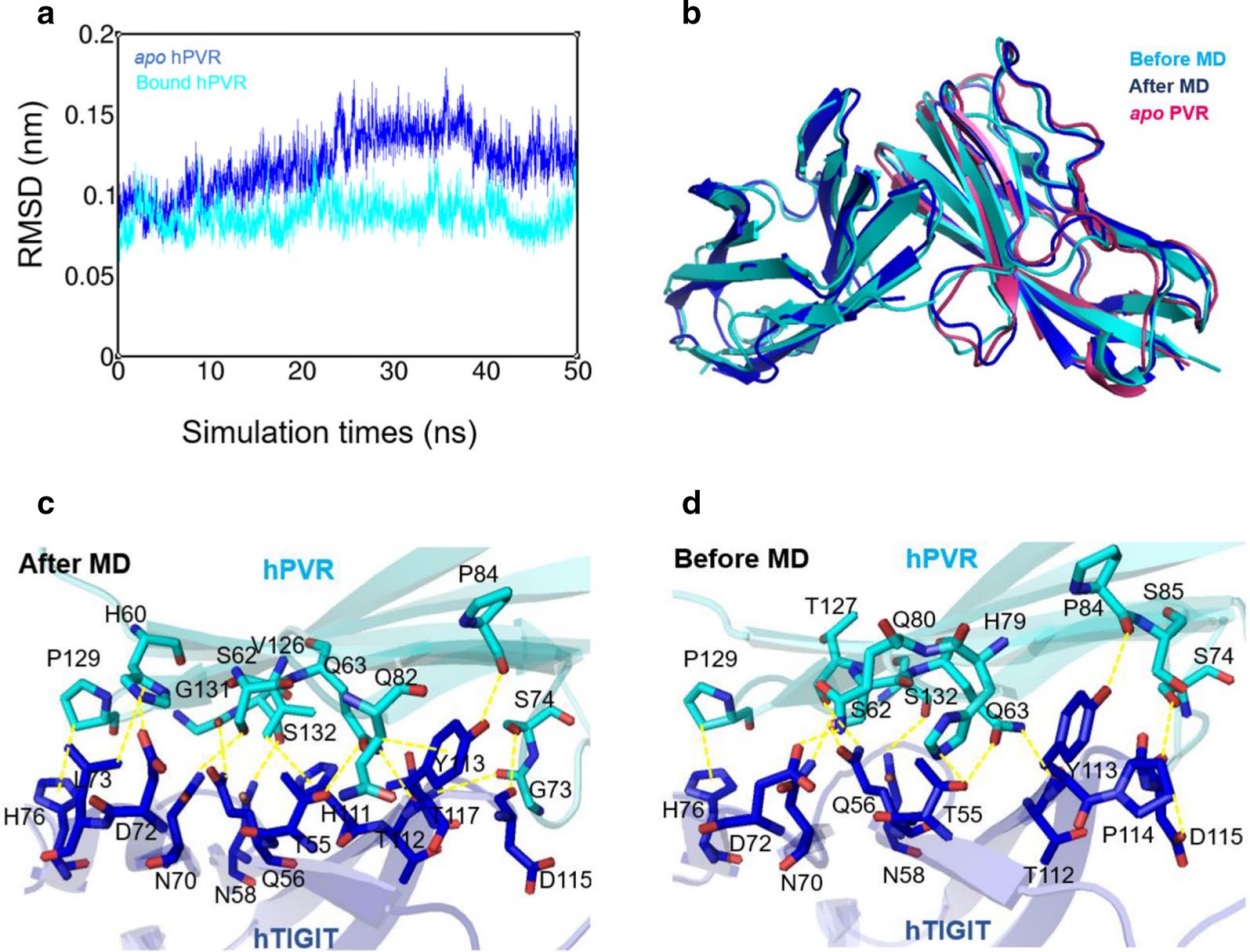
Structural fluctuations and hydrogen bond network during molecular dynamics simulations. **a** The root mean square deviation (RMSD) of hPVR in *apo* state (cyan) and in bound state (blue) were considerably stable during MD simulations. The structural fluctuation of the former was slightly larger than that of the latter and the RMSD score remained 0.05 to 0.2 nm. **b** The averaged structure of hTIGIT/hPVR complex (After MD, show in blue) and hPVR in *apo* state (*apo* hPVR, show in warmpink) during the course of simulations, which were aligned to the TIGIT/PVR complex without MD simulations (Before MD, show in cyan). (**c**, **d**) Hydrogen bond network between hPVR (cyan) and hTIGIT (blue) in two states were represented by dashed lines

The structural properties of TIGIT/PVR complex and PVR bound to poliovirus were summarized (Table 1), and crystal structure of 3UDW was chosen based on the resolved resolution, R-value, and mutation sites introduced in the crystal structure to study the binding of PVR and TIGIT. To further elucidate the interaction between human PVR and human TIGIT as well as structural dynamics at the atomic level, 50-ns MD simulations were performed for two systems (hPVR *apo* and hPVR bound states) under the physiological conditions in which the effects of force field, water, temperature and pressure were well considered. The root mean square deviation (RMSD) calculations were monitored during MD simulations. The RMSD curves of the two trajectories gradually reached to the equilibrium state, indicating that the PVR molecules attained a structurally stable state (Fig. 1a). Therein, the PVR in the bound state showed few fluctuations than PVR in the *apo* state, indicating that the conformation of PVR in complexed with TIGIT was more stable than that in *apo* state (Fig. 1a). The PVR in the *apo* state fluctuated greatly in 30–40 ns and then reached a stable state, which implied that the PVR in the *apo* state went through momentous structural rearrangements during the MD simulations (Fig. 1a). The averaged structure after 50-ns MD simulations was superimposed to the relevant crystal structure (PDBID: 3UDW) (Fig. 1b), and the results indicated that there were more obvious changes on the loops near the TIGIT binding interface, but not the beta-sheets at the interface between PVR and TIGIT (Fig. 1b). Hereafter, the interaction networks between crystal structure and the averaged structure were analyzed to study whether the MD simulations could indicate more potential residues for TIGIT interaction (Fig. 1c, d). Residues S62, Q63, S74, H79, Q80, P84, S85, T127, P129, S132 in PVR protein were involved in TIGIT binding based on the crystal structure, while the contact residues in PVR were H60, S62, Q63, G73, S74, Q82, P84, V126, P129, G131, S132 after the MD simulations (Fig. 1c, d). The MD averaged conformation showed that residues H60, G73, Q82, V126 and G131 in PVR generated hydrophobic interaction to TIGIT, which were not observed from the crystal structure.

### Atomic dynamics of PVR in different states

**Fig. 2:**
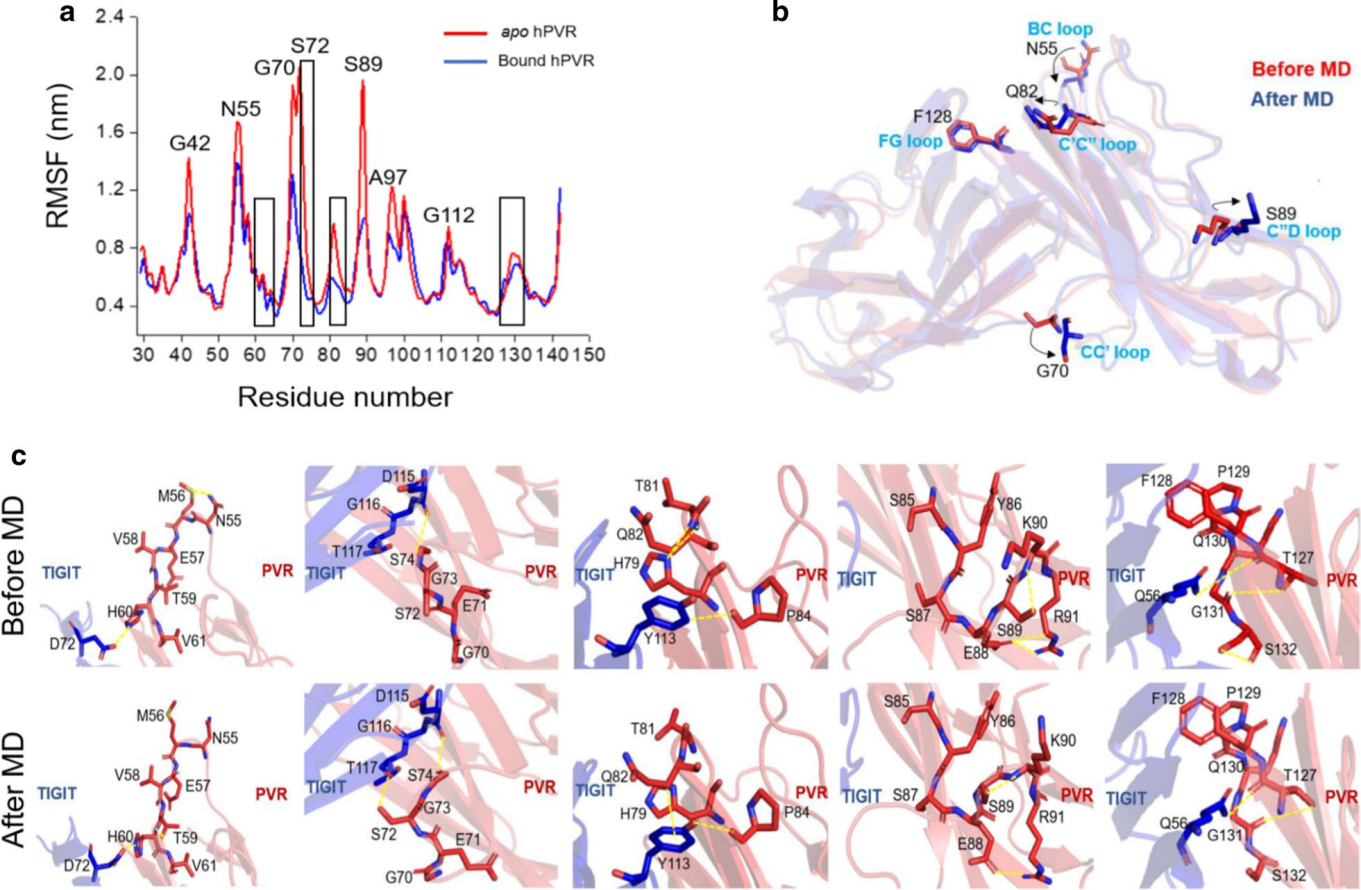
Comparison of atomic fluctuations between apo and bound hPVR. **a** The root mean square fluctuations (RMSF) of per residue describe that the hPVR in bound state remained more rigid, while the hPVR in *apo* state was mostly flexible. The black box showed the residues on the interface of hTIGIT/hPVR complex. **b** The superimposed sturctures of hTIGIT/hPVR complexes after MD simulations (After MD, show in blue) and without MD simulations (Before MD, show in red), where the residues with large fluctuations was indicated. **c** The state of residues near the five regions (BC loop, CC′ loop, CC″ loop, C″D loop and FG loop) was compared, of which the RMSF value was significantly influenced

The residual fluctuations of PVR in *apo* and ligand bound states were analyzed. During the 50-ns MD simulations, root mean square fluctuation (RMSF) values of each residue were calculated and the results indicated that the residues in *apo* PVR was more flexible than that in the TIGIT bound PVR (Fig. 2a). The RMSF values of the residues such as G42, N55, G70, S72, and S89 were significantly different between the *apo* and bound state, and these residues, especially N72, were more flexible in the *apo* state (RMSF > 2 Å) than in bound state (RMSF < 0.8 Å) (Fig. 2a). The residues at the interface were rigid and the residues at PVR BC loop, CC′ loop, CC″ loop, C″D loop, and FG loop which were adjacent to the interface were relatively more flexible (Fig. 2a and Additional file 1: Fig. S1d). The comparison between *apo* PVR and TIGIT bound PVR indicated that the orientations of five residues N55, G70, Q82, S89, and F128 in PVR changed at different states (Fig. 2b). MD simulations revealed that the regions including these five residues were in loop motifs (such as CC′ loop, C′C″ loop, and FG loop) and the five regions which were relatively close to the binding interface underwent a large intra-molecular rearrangement to facilitate the TIGIT binding process. Meanwhile, the hydrogen bonds formed in these regions, such as BC loop and C″D loop, were less stable and might be broken to allosterically regulate the conformation of hPVR to interact with TIGIT (Fig. 2c).

Therefore, the residues H60, S72, S74, Q82, P84, and T127 in PVR that are involved in forming the interactions with TIGIT in the loop regions including BC, CC′, C′C″, and FG loop which were adjacent to the binding interface were selected as potential sites for in silico mutagenesis. The residues V61, G73, H79, S85, G131, and S132 which located close to the TIGIT binding residues were also selected as potential sites for in silico mutagenesis. We proposed that mutagenesis at these residual sites were most probably able to improve the binding affinity to TIGIT.

### Distance variations of residue pairs during simulations

**Fig. 3:**
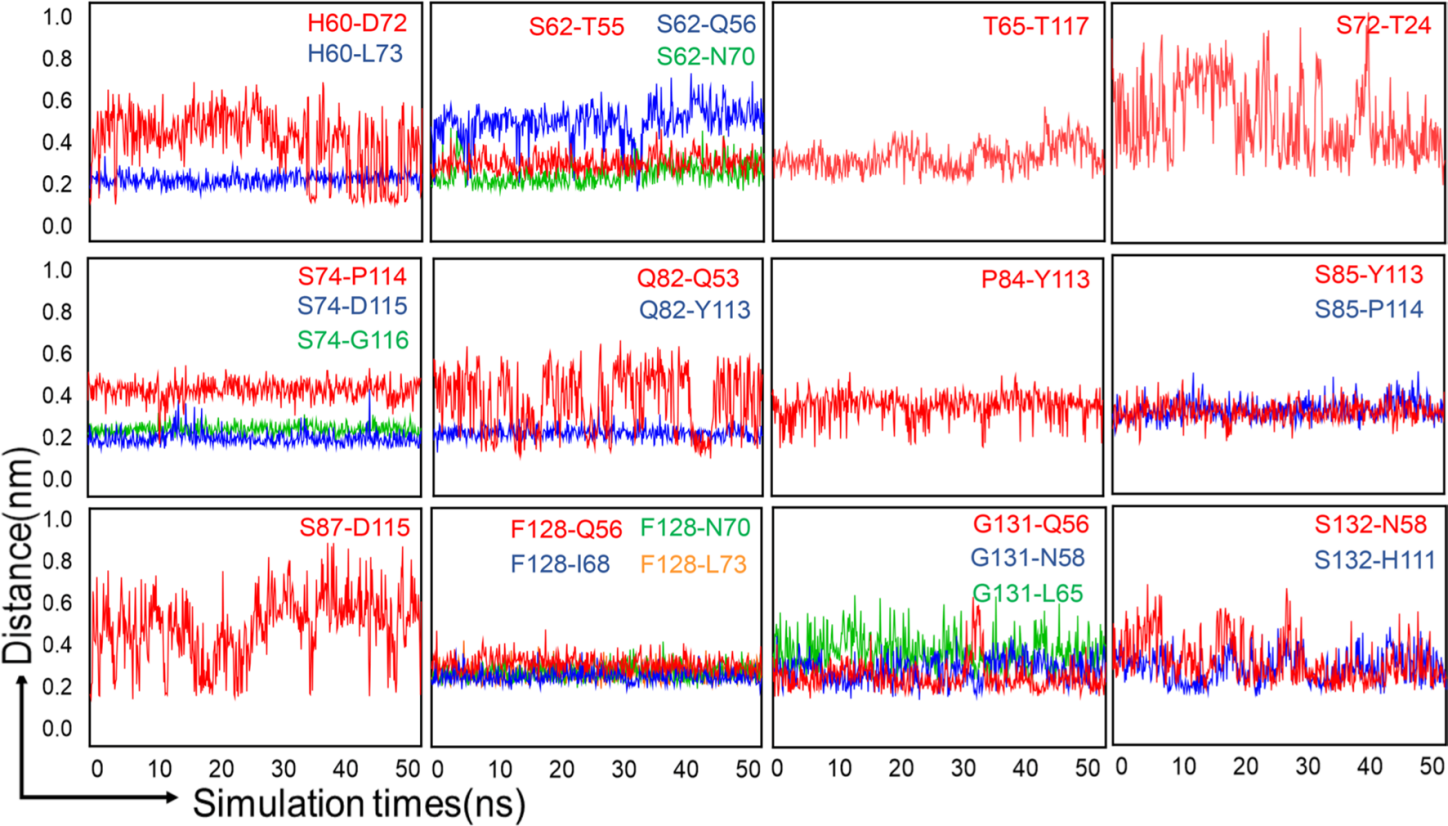
The distance variations of key residue pairs for hTIGIT/hPVR complex. Graphs showed the distance changes of residue pairs forming hydrogen bonds and Van der Waals distance interactions in TIGIT/PVR complex during MD simulations. The residues in the hPVR are written in the front of the residue pair, the residues in the hTIGIT are written in the back

In order to study the residues that are involved in the process of TIGIT/PVR interactions, we measured the distance changes of the amino acid pairs which either contributed to hydrogen bond formation or Van der Waals interactions during MD simulations. The residue in the hPVR was labelled in the front of the residue pair, and the residue in the hTIGIT was marked in the back. During 50-ns MD simulations, the distance of a few amino acid pairs changed slightly and stayed in a relatively stable state, and large fluctuations have been detected in some amino acid pairs (Fig. 3). The residue pairs for the interactions which were crucial for TIGIT binding had a stable distance during the MD simulations (such as _PVR_S74-_TIGIT_P114/D115/G116, _PVR_S85-_TIGIT_Y113/P114 and _PVR_F128-_TIGIT_Q56/I68/N70/L73) (Fig. 3). The distances between the residual pairs (such as _PVR_H60-_TIGIT_D72, _PVR_S62-_TIGIT_Q56, _PVR_T65-_TIGIT_T117, _PVR_S72-_TIGIT_T24, _PVR_Q82-_TIGIT_Q53, _PVR_P84-_TIGIT_Y113, _PVR_S87-_TIGIT_D115, _PVR_G131-_TIGIT_L65, _PVR_S132-_TIGIT_N58, and _PVR_S132-_TIGIT_H111) exhibited large fluctuations. Considering that the residues with small fluctuations may be crucial for the protein–protein interaction, we decided to mutate the residues with relatively larger fluctuations in PVR in order to facilitate the formation of more stable amino acid pairs with TIGIT (Fig. 3). The residues S72 and S87 on PVR only possessed one interaction to TIGIT and the distance for these interactions varied greatly during MD simulations, therefore we proposed that mutations of the amino acids S72 and S87 of PVR could increase the binding affinity between PVR and TIGIT (Fig. 3). We finally selected the residues with large distance variations for designing the high affinity PVR mutants which may interrupt the formation of wild type of PVR and TIGIT.

### hPVR mutants generated by the in silico mutagenesis

The residues of PVR with potential values obtained by different analysis methods described above were summarized and used to perform virtual alanine scanning and residue mutagenesis. Almost all residues that mutated to alanine (Additional file 2: Table S1) reduced the affinity of PVR with TIGIT and decreased the stability of the protein, which proved that these residues were beneficial to the binding of PVR to TIGIT. Then, these residues were mutated into 20 natural amino acids and 3600 PVR mutants were obtained. The binding energies of PVR mutants to TIGIT were calculated by MM/GBVI scoring function in MOE package. Residues substitution of PVR in the “key” region of PVR ^127^TFP^129^ [41] were not conducive to the improvement of binding affinity and protein stability. However, amino acids replacement of G131 and S132, near the “key” region of PVR ^127^TFP^129^, S72, S74, S87 with large distance variations (Fig. 3) and residual dynamics (Fig. 2a) had greater potential to enhance the binding affinity. According to the binding affinity, protein stability and residual fluctuations during MD simulations, 10 mutants S87W, S72W, S72R, T65E, G131W, S74W, S132R, G131M, G131V and S132Q (Table 2) were selected for subsequent measurement of biological functions.

**Table 2:** Design of PVR mutants based on key residue positions

No.	Mutants	Δ Affinity (kcal/mol)	Δ Stability (kcal/mol)
1	S87W	− 6.0533	− 0.4876
2	S72W	− 5.8471	− 0.0612
3	S72R	− 5.8053	0.2568
4	T65E	− 4.7483	1.0379
5	G131W	− 4.6440	− 0.1364
6	S74W	− 4.5593	0.0172
7	S132R	− 4.5511	0.2703
8	G131M	− 4.4606	− 0.3685
9	G131V	− 4.2638	− 0.8332
10	S132Q	− 4.2539	0.5422

### Binding analysis between hPVR mutants and hTIGIT

Cell-based flow cytometry analysis was carried out to measure the binding affinity of PVR mutants with TIGIT. Ten single-point mutations of PVR including S87W, S72W, S72R, T65E, G131W, S74W, S132R, G131M, G131V, and S132Q were constructed and the full-length proteins were expressed in CHO-K1 cells, respectively. We performed monoclonal screening after constructing stable cell lines of mutants. Cell lines expressing basically consistent PVR mutants were selected for this study (Additional file 1: Fig. S2a). It was found that the mutant T65E merely expressed in a small amount on CHO-K1 cells (Additional file 1: Fig. S2a). Total proteins of the cells stably expressing PVR mutants were extracted, and western blotting was used to detect the expression of PVR protein (Additional file 1: Fig. S2b), which were comparative to those obtained by flow cytometry (Additional file 1: Fig. S2a). The PVR antibody used for western blotting is recombinant fusion protein containing a sequence combining with amino acids 220–345 of human PVR, which indicated that mutations in the residues of PVR D1 domain (corresponding to amino acids 30–143) do not affect the detection of PVR expression by PVR antibodies. These results implied that mutant T65E affected PVR expression.

**Fig. 4:**
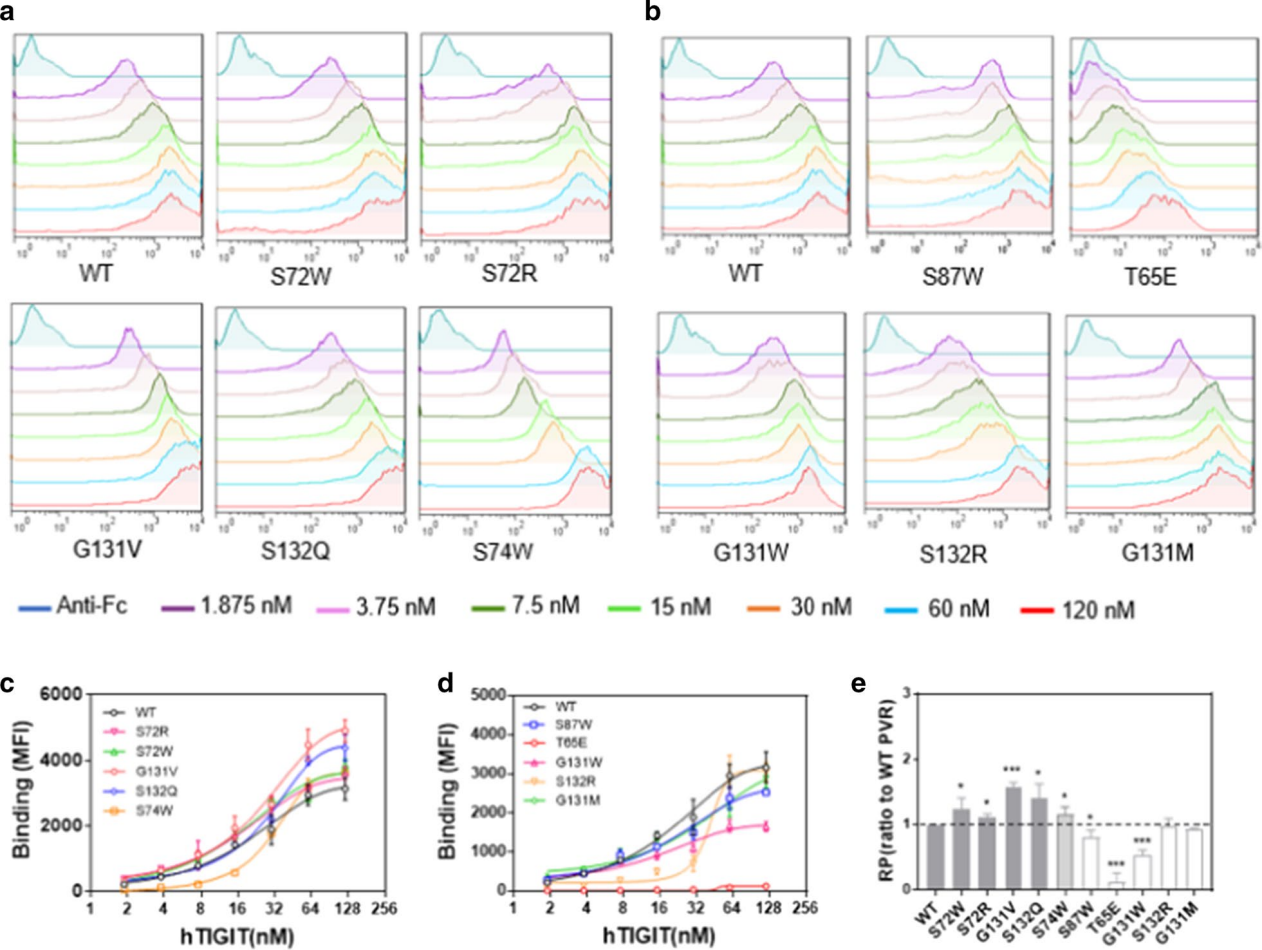
The binding affinity of hPVR mutants with hTIGIT. **a**, **c** Parental CHOK1 cells as well as CHOK1 cells overexpressing WT hPVR and mutant hPVR were used in binding assays. The binding of WT and mutant hPVR with hTIGIT-Fc were assessed by flow cytometry using an anti-human Fc antibody. Representative curves of three independent measurements were shown. **b**, **d** The binding affinity between hPVR mutants and hTIGIT-Fc at various concentrations from 120 to 1.875 nM. Graphs showed mean ± standard error of the mean (SEM) of three independent experiments. **e** The binding affinity of hPVR mutants were normalized *versus* WT hPVR (dashed line) at the concentration of 120 nM, which was defined as relative hTIGIT binding potency (RP) values of the hPVR mutants. *P < 0.05, **P < 0.01 and ***P < 0.001 by Student’s *t-*test

After the successful construction of the PVR mutant cell lines, we used flow cytometry to measure the binding affinity of PVR mutants (S87W, S72W, S72R, T65E, G131W, S74W, S132R, G131M, G131V or S132Q) to TIGIT-Fc eukaryotic protein. The concentrations of eukaryotic human-TIGIT-Fc were 1.875 nM, 3.75 nM, 7.5 nM, 15 nM, 30 nM, 60 nM and 120 nM (Fig. 4a, c). The mutations of S72R, S72W, G131V, S132Q and S74W significantly enhanced the binding of PVR-ecto mutants to TIGIT (Fig. 4a, b).

The mutant G131V significantly enhanced the binding affinity to TIGIT because of the site was replaced by hydrophobic side chain (Fig. 4b). The mutant S132Q presented higher binding affinity with TIGIT and Glutamine (Gln) had a longer polar uncharged side chain than serine (Ser), which indicated that lower steric hindrance performed a crucial role in the interaction between PVR and TIGIT (Fig. 4b). It was surprising that the mutants S72R and S72W which changed the polar uncharged Ser (serine) to positively charged Arg (arginine) or hydrophobic Trp (tryptophan) with aromatic group side chains slightly increased their affinity with TIGIT (Fig. 4b). However, the corresponding PVR-ecto mutants which introduced S87W, T65E, G131W, S132R or G131M mutation decreased the binding affinity to TIGIT (Fig. 4c, d). However, mutant T65E exhibited relatively weak binding capacity to TIGIT (Fig. 4d), which might be caused by the low expression level. The relative binding affinity of each mutants was standardized by the binding affinity of the wild type PVR at the concentration of 120 nM (Fig. 4e). The correlation between the binding affinity and the protein expression level of PVR mutants was studied and the P values were greater than 0.05 and R^2^ were less than 0.35 under different TIGIT protein concentrations (Additional file 1: Fig. S3). The results showed no correlation between the binding affinity and the expression level of PVR mutants, which indicated that the binding affinity of the PVR mutants were mainly depended on the actions of the mutations.

To further verify that there was no correlation between the binding affinity and protein expression, additional experiment was conducted. Firstly, the membrane protein of PVR mutants fused with EGFP were extracted and quantified by the fluorescence value of EGFP to exclude the influence of protein expression on binding affinity. Then the binding affinity of PVR mutants with TIGIT-His eukaryotic protein was identified by microscale thermophoresis (MST) to calculate the K_D_ values of PVR mutants. It was shown that the binding affinities of mutants _PVR_G131V (K_D_ = 0.016 μM) and _PVR_S132Q (K_D_ = 0.058 μM) to TIGIT-His were enhanced about 72-fold and 20-fold, respectively (Additional file 1: Fig. S4a, d-e) compared with wild-type PVR (K_D_ = 1.146 μM). The mutants _PVR_S72W (K_D_ = 0.328 μM) and _PVR_S72R (K_D_ = 0.549 μM) increased their binding affinity to hTIGIT by approximately 4 and 2 times (Additional file 1: Fig. S4a-c). The results were consistent with that obtained through flow cytometry methods (Additional file 1: Fig. S4a-e, Fig. 4a, c). Also, the mutants _PVR_S74W, _PVR_S87W, _PVR_T65E, _PVR_G131W, _PVR_S132R, and _PVR_G131M with lower binding affinity consistently impaired the binding (Additional file 1: Fig. S4a, f-k). It was worth mentioned that the fluorescence value of the extracted membrane protein of mutant _PVR_T65E was almost undetectable which was consistent with the previous results of protein expression (Additional file 1: Fig. S2) by flow cytometry and western blotting and it was difficult to detect the binding affinity between these two proteins through MST.

### High affinity hPVR mutants decreased IL-2 production

**Fig. 5:**
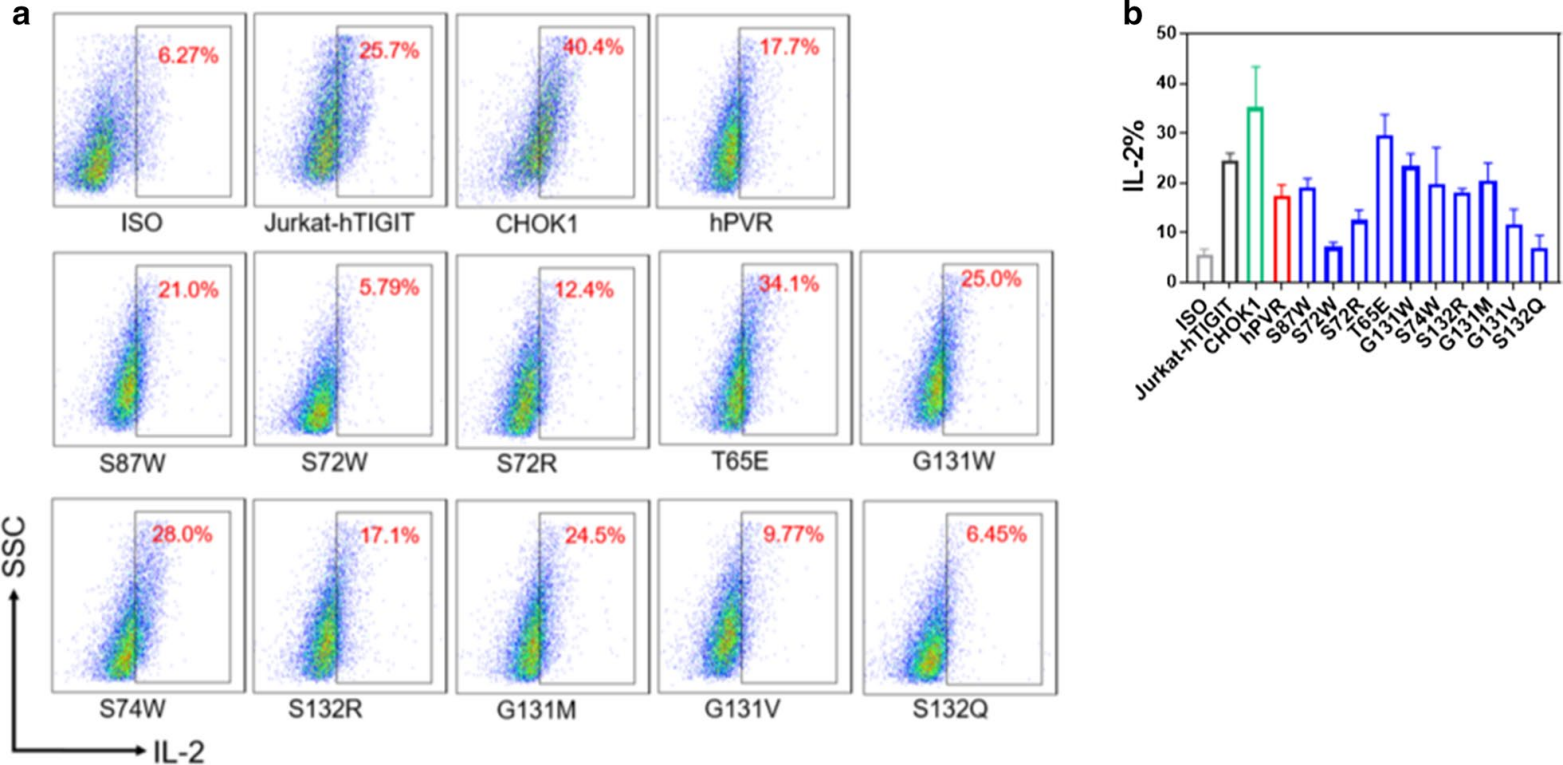
IL-2 production of Jurkat-hTIGIT cells co-cultured with hPVR mutants. **a** Jurkat cells overexpressing hTIGIT were co-cultured with CHOK1, CHOK1-hPVR, CHOK1-mutants for 48 h, which was stimulated with 1 μg/mL human anti-CD3 and 0.5 μg/mL human anti-CD28. Protein transport inhibitor was added in the last 4 h. The frequency of IL-2-secreting Jurkat-hTIGIT cells were detected by flow cytometry. Data were representatively independent of three measurements. **b** Analysis of the frequency of IL-2-secreting was shown. Graphs showed mean ± standard error of the mean (SEM) of three independent experiments

To further explore whether PVR mutants affect the biological function, we transfected human TIGIT into Jurkat cells which lacked endogenous TIGIT expression and co-cultured Jurkat-TIGIT cells with CHOK1 cells that expressing PVR Wild-Type or mutants. The expression of wild type PVR on CHOK1 cells reduced the proportion of Jurkat-hTIGIT cells which produced IL-2 compared to the parental CHOK1 cell line by using flow cytometry analysis (Fig. 5a, b). The mutants _PVR_S72W, _PVR_S72R, _PVR_G131V, and _PVR_S132Q induced more potent inhibitory effects of PVR on TIGIT, resulting in a remarkable reduction in the proportion of Jurkat-hTIGIT cells producing IL-2 compared to Jurkat-TIGIT cells co-cultured with wild-type PVR (Fig. 5b), which was consistent with previous results that these mutants showed higher affinity with TIGIT (Fig. 4a, b). However, _PVR_S87W, _PVR_T65E, _PVR_G131W, _PVR_S74W, _PVR_S132R, and _PVR_G131M exhibited a reduced inhibitory effect on IL-2 secretion, and the proportion of Jurkat-TIGIT cells that produced higher level of IL-2 compared to wild-type PVR (Fig. 5b). Among them, _PVR_S87W, _PVR_T65E, _PVR_G131W, _PVR_S132R, and _PVR_G131M had a weakened inhibitory effect on TIGIT, which were consistent with the results of the aforementioned binding affinity on TIGIT (Fig. 4a, b). The mutant _PVR_S74W slightly reduced inhibitory effect on TIGIT compared with wild-type PVR, but there was no significant difference (Fig. 5b). The previous affinity results showed that the binding affinity of _PVR_S74W with TIGIT was lower than that of wild-type PVR at low TIGIT-Fc concentrations, but the affinity of _PVR_S74W with TIGIT was slightly higher than wild-type PVR when TIGIT-Fc was almost reached the saturated concentration (120 nM) (Fig. 4b). Therefore, the effect of _PVR_S74W on TIGIT was not different from that of wild-type PVR in the co-culture experiment (Fig. 5b), which might due to the reason that the concentration of PVR interacting with TIGIT did not reach the saturated state. Surprisingly, the co-culture of Jurkat-hTIGIT and CHOK1 cells secreted higher level of IL-2 than that secreted by Jurkat-hTIGIT cells alone (Fig. 5a, b), which might be caused by some stimulatory molecules expressing on CHOK1 cells.

## Discussion

TIGIT is emerging as a critical immune checkpoint that has been involved in regulating immune effector function. Overexpression of PVR on cancer cells can restrain T cell and NK cell responses because PVR can negatively manipulate TIGIT functions [26, 27, 42]. The structural properties of TIGIT/PVR complex were well studied so far and several antibody drugs targeting TIGIT/PVR pathway have been developed [33, 34, 41]. Studying the dynamic characteristics and binding mechanism at the atomic level between TIGIT and PVR will contribute to the rational discovery and design of PVR inhibitors such as high affinity PVR protein, peptides and small molecules.

The crystal structures of TIGIT and PVR (Table 1) provide atomic details to understand the coordinates of the PVR and the binding mode of the complex, but they lack the dynamic information about the protein. In order to deeply study the flexibility and fluctuation of PVR in different states, we analyzed the overall and atomic level fluctuations of PVR by using MD simulations. The distance variations of important residue pairs for TIGIT/PVR complex revealed that the residue pairs formed by H60 and S62 on the C sheet (near the BC loop) of PVR, Q82 on the C loop of PVR, S87 on the C″D loop of PVR, and G131 and S132 on the FG loop of PVR underwent large conformational movements (Fig. 3). This is consistent with the results of atomic fluctuations for the corresponding residues (Fig. 2a, c) during MD simulations. Most of these residues with the distance between 4.5 and 6 Å to hTIGIT molecule are located in the loop region and fluctuated greatly, which suggested that the residues in the loop region play a crucial role for TIGIT/PVR interaction.

The crystal structure of TIGIT bound to PVR revealed that two motifs (^112^TYP^114^ in TIGIT and ^127^TFP^129^ in PVR) played a crucial role in the interaction of TIGIT and PVR [41]. The “key” residues Y113 in TIGIT and F128 in PVR were respectively inserted into a hydrophobic pocket formed by the engaging molecule. The mutation analysis of the TIGIT/PVR interaction revealed that substitution of Q63 in the (V/I) (S/T) Q motif and F128 in the ^127^TFP^129^ motif with alanine in PVR reduced the binding affinity with TIGIT [41]. To obtain the energy contribution of the residues in PVR to the TIGIT/PVR interaction, we analyzed some hotspot residues in PVR through molecular dynamics simulation analysis and then performed in silico mutagenesis on hotspot residues. Among them, mutation of Q63 and F128 in PVR to Ala (alanine) affected the binding of TIGIT to PVR and the structural stability of TIGIT/PVR complex, which was consistent with the results of mutation analysis of the TIGIT/PVR interaction. In addition, H60 and V126 on PVR were important to the interaction between TIGIT and PVR, while other hot spot residues but not G131 showed little effects on TIGIT/PVR interaction. The G131A mutant in PVR was not conducive to the binding of TIGIT to PVR, which might be owing to that the steric hindrance generated by the side chain of alanine which affected the function of the “key” region of PVR ^127^TFP^129^. In this study, hotspot residues were considered as potential candidates for designing high affinity PVR which required an in silico virtual screening and in vitro binding affinity verification.

The conformation of PVR was dynamically changed to facilitate TIGIT interaction. The loops in the protein played critical roles in complex formation. We identified several residues on the TIGIT binding interface or adjacent TIGIT/PVR interface which formed new interface during the MD simulations. These residues mainly located in the loop region near the binding interface and can be identified as potential candidates for mutagenesis in the design of high affinity PVR mutants. To prove our hypothesis, we obtained PVR mutants at these sites and then expressed the mutants on CHOK1 cells. Some mutants (_PVR_S72W, _PVR_S72R, _PVR_G131V, and _PVR_S132Q) in the CC′ and FG loop located close to the interface enhanced the binding of PVR to TIGIT. Surprisingly, it was found that _PVR_T65E affected the expression of PVR, which might be due to the fact that charged Glu (glutamate) affected the stability of the molecular structure of PVR. These results are in line with our hypothesis that substitution of residues in the loop region closer to the interface contribute to TIGIT/PVR interaction, and mutations at the interface are not beneficial to obtain high affinity mutants.

TIGIT, DNAM-1 and CD96 could recognize nectin and nectin-like adhesion molecules, such as necl-5 (PVR) and nectin-2. These molecules play important roles for NK and T cell function [26, 27, 42–45]. The binding of TIGIT to PVR (K_D_ = 3.2 ± 0.4 μM) [20] was stronger than it to nectin-2 (K_D_ = 5.8 ± 0.6 μM) [20] or CD96/PVR (K_D_ = 10.2 ± 1.1 μM) [46], which highlights the importance of the TIGIT/PVR pathway in immune regulation. We acquired several high affinity mutants of PVR, which have increased binding affinity to TIGIT and may serve as potential drugs in targeting the TIGIT related pathway. However, the PVR mutants may also bind to other molecules such as TIGIT, DNAM-1 and CD96, therefore the specificity of the mutants should be further considered.

Antibodies targeting TIGIT/PVR pathway have achieved good effects in cancer treatment. The antibody targeting TIGIT using alone or combing other antibodies has achieved significant anti-tumor effects [27]. However, the poor tissue penetration and Fc-effector functions limit the development of antibody drugs in cancer treatment. With relatively small molecular weight, PVR mutants without Fc segment might be utilized as protein drugs for cancer treatment, which needs further researches to test the anti-tumor effects of the mutant proteins both in vitro and in vivo. Also, high affinity PVR mutants might be used in combination with multispecific drugs targeting multiple immune pathways to improve the anti-tumor effects.

In summary, we provided a dynamic model for PVR at two different states by applying MD simulations. We also identified several high affinity PVR mutants by using in silico mutagenesis and cell assays. Four PVR mutants (_PVR_S72W, _PVR_S72R, _PVR_G131V and _PVR_S132Q) enhanced binding affinity of PVR to TIGIT and induced more potent inhibitory effects on TIGIT overexpressed Jurkat cells.

## Conclusion

Molecular dynamics simulations and in silico mutagenesis were applied to study the dynamics of PVR in both states and screen a list of high affinity mutants. The binding affinity of the mutants were measured by cell assay with FACS. Four mutants (_PVR_S72W, _PVR_S72R, _PVR_G131V and _PVR_S132Q) with enhanced affinity for TIGIT could induce more potent inhibitory effects on TIGIT overexpressed Jurkat cells, which could contribute to design new candidates for TIGIT-targeting therapies.

## Supplementary information

**Additional file 1**: **Figure S1.** Protein topology of hTIGIT and hPVR, along with the structure of TIGIT/PVR complex. (a, b) hTIGIT and hPVR contained ectodomain (blue), transmembrane domain (red) and intracellular domain (purple). The glycosylation sites of the extracellular domain were marked with a yellow ball. (c, d) The residue sequences of hTIGIT and hPVR, where the secondary structure α-helix and β-sheet were noted by cyan and yellow shadow respectively, and the residues forming the disulfide bond were represented by red. (e) Model of hTIGIT complexed with hPVR, which was retrieved from heterotetrameric hTIGIT/hPVR complex (PDB ID code 3UDW). The secondary structure and disulfide bond were shown in the same way as above. **Figure S2.** Stable cell lines of hPVR mutants. (a) CHOK1 cells overexpressing WT and mutant hPVR were stained with anti-human PVR APC. For each histogram, the filled blue histogram with blue line is the hPVR specific antibody and the histogram with red lines is the isotype control. (b) Lysates of CHOK1, CHOK1-hPVR, and CHOK1-mutants were used for western blotting. The blot was developed by chemiluminescence. **Figure S3.** Relationship between protein expression and binding affinity. Protein expression were normalized versus wild type hPVR cells. Graphs showed the correlation between protein expression and binding affinity When hTIGIT-Fc at different concentrations. The Pearson correlation coefficient and P were shown.** Figure S4.** Binding affinity of PVR mutants fused with EGFP to hTIGIT-His. The membrane protein of PVR mutants fused with EGFP was used to detect the binding affinity with hTIGIT-His. The concentration of TIGIT-His was serially diluted by two-fold with 0.000153 μM from 25 μM. The KD values of PVR mutants with hTIGIT-His were shown. Graphs were representative of three independent experiments..

**Additional file 2**: **Table S1.** Alanine scanning of important residue positions.

## Data Availability

The datasets used and/or analyzed during the current study are available from the corresponding author on reasonable request.

## Abbreviations

TIGIT: T cell immunoglobulin and immunoreceptor tyrosine-based inhibitory motif domain
hTIGIT: Human TIGIT
PVR: Poliovirus receptor
hPVR: Human PVR
MD: Molecular dynamics simulations
MOE: Molecular operating environment
EM: Energy minimization
PBC: Periodic boundary conditions
RMSD: Root mean square deviation
RMSF: Root mean square fluctuation
_PVR_S72W: The mutation of S72W in PVR
