# Supplementary material for: Computer-aided design of PVR mutants with enhanced binding affinity to TIGIT
Source: Cell Commun Signal. 2021 Feb 8;19:12. doi: 10.1186/s12964-020-00701-y (PMC7869511; doi:10.1186/s12964-020-00701-y)
Supplement: Supplementary file 2 — Additional file 1: Figure S1. Protein topology of hTIGIT and hPVR, along with the structure of TIGIT/PVR complex. (a, b) hTIGIT and hPVR contained ectodomain (blue), transmembrane domain (red) and intracellular domain (purple). The glycosylation sites of the extracellular domain were marked with a yellow ball. (c, d) The residue sequences of hTIGIT and hPVR, where the secondary structure α-helix and β-sheet were noted by cyan and yellow shadow respectively, and the residues forming the disulfide bond were represented by red. (e) Model of hTIGIT complexed with hPVR, which was retrieved from heterotetrameric hTIGIT/hPVR complex (PDB ID code 3UDW). The secondary structure and disulfide bond were shown in the same way as above. Figure S2. Stable cell lines of hPVR mutants. (a) CHOK1 cells overexpressing WT and mutant hPVR were stained with anti-human PVR APC. For each histogram, the filled blue histogram with blue line is the hPVR specific antibody and the histogram with red lines is the isotype control. (b) Lysates of CHOK1, CHOK1-hPVR, and CHOK1-mutants were used for western blotting. The blot was developed by chemiluminescence. Figure S3. Relationship between protein expression and binding affinity. Protein expression were normalized versus wild type hPVR cells. Graphs showed the correlation between protein expression and binding affinity When hTIGIT-Fc at different concentrations. The Pearson correlation coefficient and P were shown. Figure S4. Binding affinity of PVR mutants fused with EGFP to hTIGIT-His. The membrane protein of PVR mutants fused with EGFP was used to detect the binding affinity with hTIGIT-His. The concentration of TIGIT-His was serially diluted by two-fold with 0.000153 μM from 25 μM. The KD values of PVR mutants with hTIGIT-His were shown. Graphs were representative of three independent experiments.. [file 12964_2020_701_MOESM2_ESM.docx]

**Supplementary material**

**Material and methods**

**Western blotting**

Cell samples were counted and washed three times using PBS. All of cell samples were simultaneously lysed by RIPA Buffer. Lysate supernatant was mixed with 1 vol of 2×SDS loading buffer, boiled at 95°C for 8 min and used as total protein fraction. Then, protein samples and molecular weight marker (catalogue number 26619, Thermo Fisher) were loaded into separate wells and separated by SDS-PAGE and transferred to a rectangular polyvinylidene difluoride (PVDF) membrane. The membrane was blocked with 5% milk for 1h at room temperature and then incubated with anti-PVR (1:1000, catalogue number A5753, ABclonal) or anti-β-actin (1:1000, catalogue number AC026, ABclonal) and gently shaken at 4°C overnight. Afterwards, the membrane was washed and incubated with HRP-conjugated secondary antibodies (1:5000, catalogue number LK2003, Sungene Biotech) at room temperature for 1 hour. Later, the membrane was washed and visualized by an ECL solution (Beyotime) and explored with C600 multifunctional molecular imaging system (Azure Biosystems) for 2 min.

**Membrane preparation of PVR mutants**

Ten single-point mutations of PVR fused with EGFP were constructed and fusion protein were expressed in HEK-293T cells. Nuc-Cyto-Mem Preparation Kit (Applygen Technologies Inc., China) were used to extract membrane protein of PVR mutants fused with EGFP. Membrane proteins were then resuspended and dissolved in phosphate buffered saline with 0.05% Tween 20 for the subsequent measurement of binding affinity by microscale thermophoresis.

**Microscale thermophoresis (MST)**

The membrane protein of PVR mutants fused with EGFP was diluted and the appropriate protein concentration was determined by adjusting its fluorescence value to 250 using microscale thermophoresis (MST) (NanoTemper technologies, GER). The His-fused protein of human TIGIT (Sino Biological Inc., China) was seriously diluted 2-fold from 25 μM to 0.000153 μM. Then the mutant sample (5 μL) from each dilution was mixed with hTIGIT-His dilution (5 μL). Finally, the samples were sucked into different capillaries by capillary phenomenon, placed into the corresponding brackets, scanned by the machine and the K_D_ value was calculated.

**Table S1. Alanine scanning of important residue positions.**

| NO. | Mutants | Δ Affinity(kcal/mol) | Δ Stability（kcal/mol） |
| --- | --- | --- | --- |
| 1 | H60A | 5.1184 | 0.6455 |
| 2 | S62A | 1.5257 | 0.4468 |
| 3 | Q63A | 5.2967 | 1.5082 |
| 4 | T65A | 0.6378 | 1.2517 |
| 5 | S72A | 0.2674 | 0.7086 |
| 6 | S74A | 0.9764 | 0.6717 |
| 7 | H79A | 1.6431 | 1.2022 |
| 8 | Q80A | 1.2123 | 1.0227 |
| 9 | Q82A | 1.7426 | 0.6891 |
| 10 | P84A | 0.1252 | 0.5648 |
| 11 | S85A | 0.4710 | 0.4474 |
| 12 | S87A | 0.0351 | 0.5034 |
| 13 | V126A | 2.1470 | 2.6023 |
| 14 | T127A | 0.1688 | 1.3854 |
| 15 | F128A | 13.4729 | 2.5663 |
| 16 | P129A | 0.2263 | 0.5971 |
| 17 | G131A | -0.4024 | 0.7763 |
| 18 | S132A | 1.2475 | 0.6345 |
