# Supplementary material for: Computer-aided design of PVR mutants with enhanced binding affinity to TIGIT
Source: Cell Commun Signal. 2021 Feb 8;19:12. doi: 10.1186/s12964-020-00701-y (PMC7869511; doi:10.1186/s12964-020-00701-y)
Supplement: Supplementary file 3 — Additional file 2: Table S1. Alanine scanning of important residue positions. [file 12964_2020_701_MOESM3_ESM.pptx]

## Slide 1
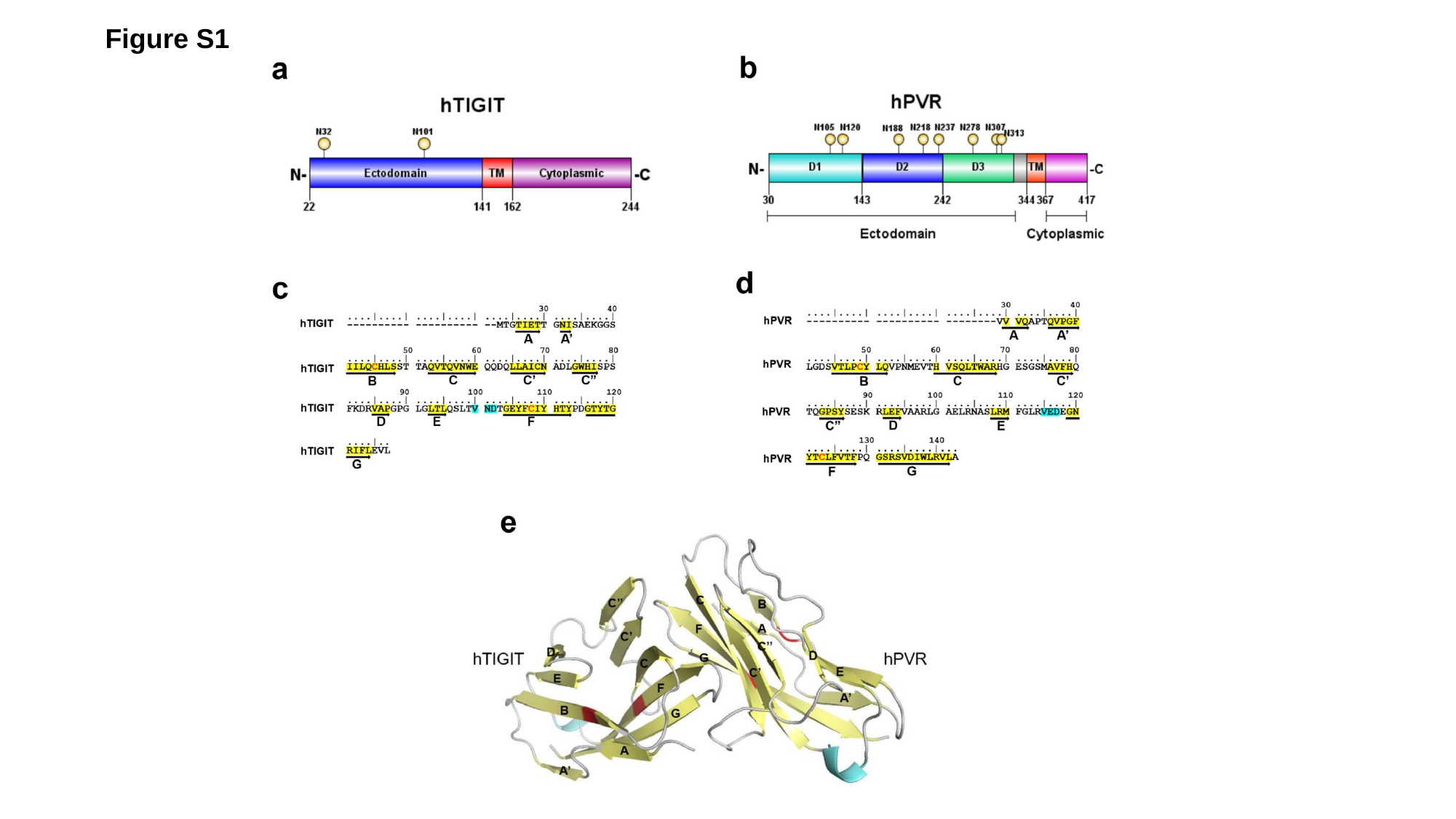

Figure S1

## Slide 2
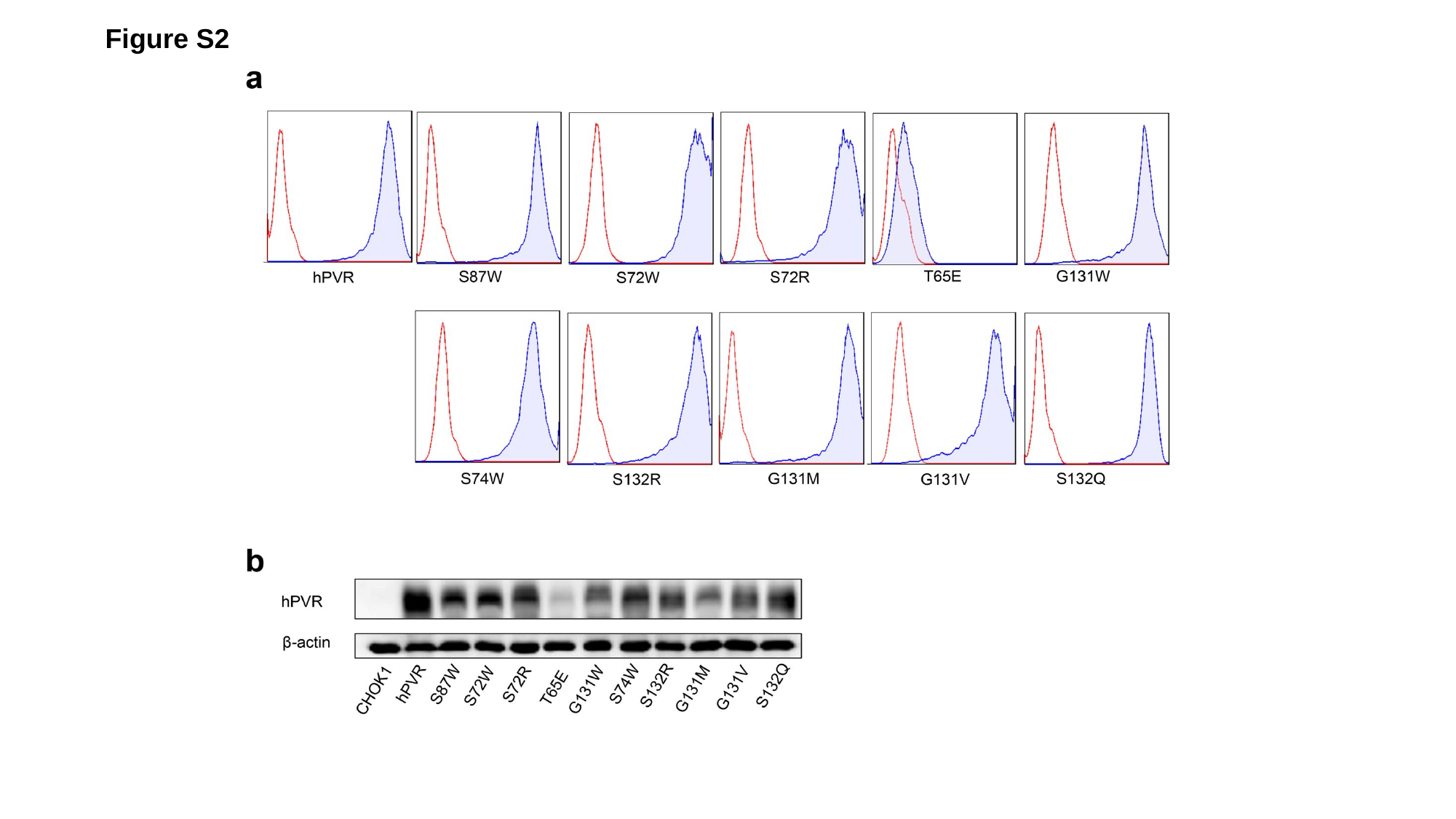

Figure S2

## Slide 3
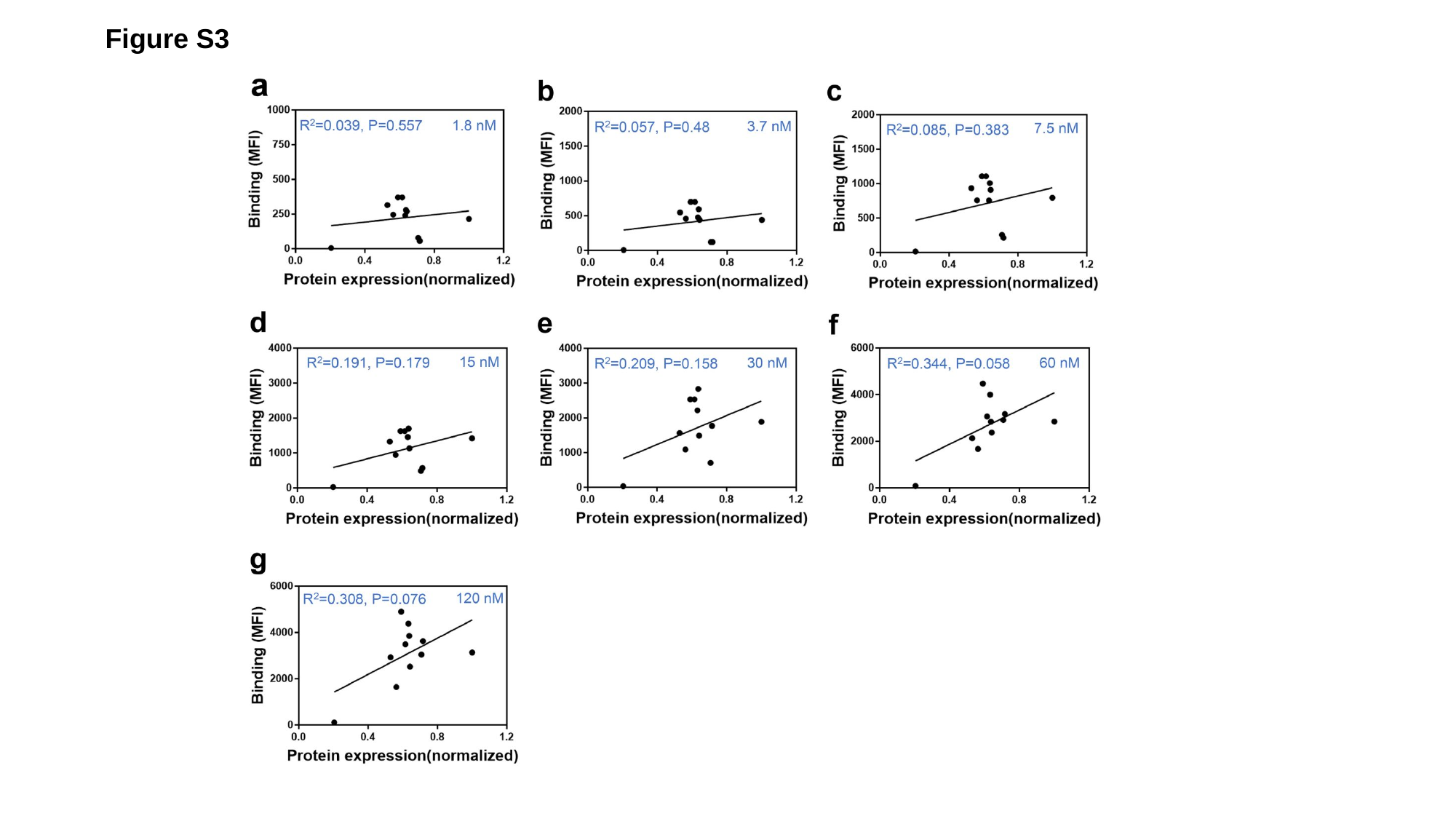

Figure S3

## Slide 4
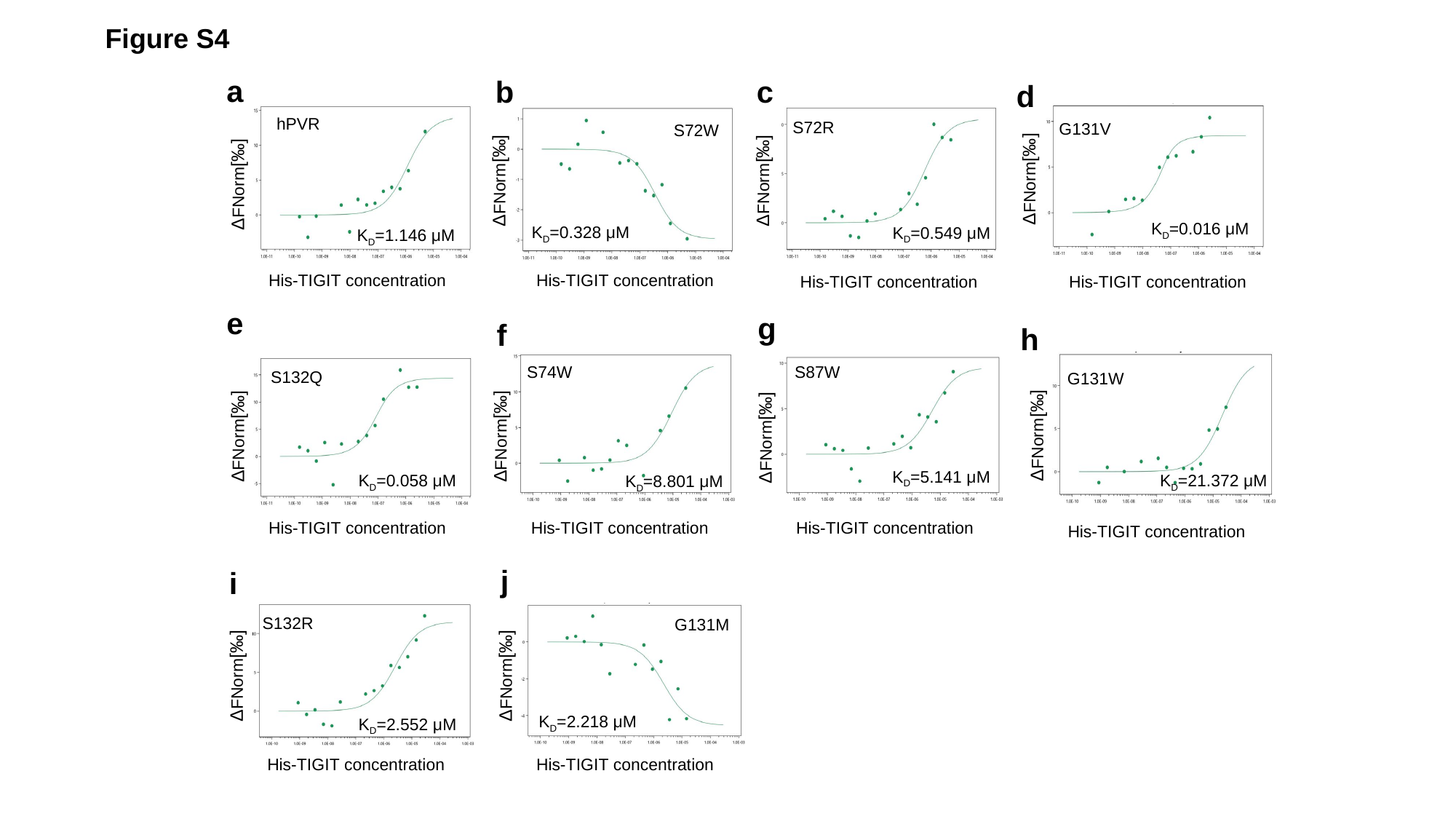

Figure S4
a
b
c
d
hPVR
S72R
G131V
S72W
ΔFNorm[‰]
ΔFNorm[‰]
ΔFNorm[‰]
ΔFNorm[‰]
KD=0.016 μM
KD=0.328 μM
KD=0.549 μM
KD=1.146 μM
His-TIGIT concentration
His-TIGIT concentration
His-TIGIT concentration
His-TIGIT concentration
e
g
f
h
S87W
S74W
S132Q
G131W
ΔFNorm[‰]
ΔFNorm[‰]
ΔFNorm[‰]
ΔFNorm[‰]
KD=5.141 μM
KD=0.058 μM
KD=21.372 μM
KD=8.801 μM
His-TIGIT concentration
His-TIGIT concentration
His-TIGIT concentration
His-TIGIT concentration
j
i
S132R
G131M
ΔFNorm[‰]
ΔFNorm[‰]
KD=2.218 μM
KD=2.552 μM
His-TIGIT concentration
His-TIGIT concentration
